# Asynchronous declines of native and exotic insect predators reduce pest suppression potential in agriculture

**DOI:** 10.1093/pnasnexus/pgag063

**Published:** 2026-03-10

**Authors:** Christie A Bahlai, Douglas A Landis

**Affiliations:** Department of Biological Sciences and Environmental Science and Design Institute, Kent State University, Kent, OH 44242, USA; Department of Entomology, Ecology, Evolution, and Behavior Program, Michigan State University, East Lansing, MI 48824, USA

**Keywords:** LTER, population dynamics, agroecosystem, insect decline, ecosystem services

## Abstract

Insect declines in agricultural landscapes have been reported as part of the larger biodiversity crisis, but long-term trends are difficult to assess due to natural population fluctuations, interactions with exotic species, and lack of consistent data. Here, we report on population trajectories of a community of predaceous lady beetles (Coleoptera: Coccinellidae) monitored annually over 31 years in a long-term agroecosystem experiment in southwestern Michigan, United States. The total lady beetle community has declined in abundance by 39% during this time, with native species exhibiting larger declines (77%) than exotics (23%). However, these gross trends mask apparent periods of stability, recovery, and, in some cases, very sharp declines lasting many years across individual species and groups. Native and exotic communities exhibit significant nonlinear abundance trends that are often asynchronous in time. Habitat perenniality moderates these patterns, with communities in annual crops exhibiting large changes and those in perennial crops following similar but less pronounced trajectories, while communities in forested habitats remain stable or exhibit gradual, nearly linear declines. Several once-common natives have fallen below detection limits, with six of 10 species not detected on an annual basis in the last 5 years. Over the full length of the study, the pest suppression potential of the entire community has declined 42%, threatening this valuable ecosystem service, which may be further undermined by the effective loss of functionally redundant species. The evidence for varying drivers of these patterns provides unique insights into the broader phenomenon of insect decline.

Significance Statement Insect population decline may not manifest in uniform patterns across regions or species. Over 30 years, both native and exotic lady beetles and the community's capacity to provide predation services have been declining in an agricultural landscape in the US Midwest. Even among these closely related organisms, rates of change and apparent drivers differ among species and across habitats in the same landscape. Selected time periods show widely varying trends illustrating the difficulty in reliably detecting population trajectories from shorter-term data sets. The overall loss of native species combined with the recent declines of several exotic species presents a serious threat to the stability of ecosystem services.

## Introduction

Insect declines are an increasingly reported phenomenon (1–4) with a variety of negative consequences (5–7) prompting multiple calls for action (8–10). Declines have been reported across multiple taxa (11–14), with time frames ranging from decades to centuries (15, 16), and with wide variation in reported drivers (17, 18). Meta-analyses of reported declines have revealed some commonalities as well as many differences in rates of change across taxa, locations, and habitats (19). However, because insects represent such a wide diversity of life histories, habitat preferences, and ecological functions, it is not surprising that studies combining data of widely divergent taxa, habitats, and sampling methods have reached different conclusions (20). Furthermore, among the literature on insect decline, even work seeking to synthesize trends is dominated by several key taxa (butterflies and bees, in particular) (18). Here, we examine a community of predacious lady beetles (Coleoptera: Coccinellidae) in a long-term agroecosystem study representing a gradient of management intensity from annual crops to forests. By combining annual sampling across multiple co-located habitat types of varying complexity, with knowledge of establishment histories and predatory capacity, we detail asynchronous phases of decline for native and exotic taxa—likely with different drivers—and the implications for species persistence and pest suppression.

Because insects are highly biodiverse, insect decline is likely a multifaceted phenomenon (21). Several publications have reported broad patterns of decline across insect taxa across disparate studies (22), loss of biomass over time (2), and loss of abundance, particularly in terrestrial insects (19). Yet, another study compiling long-term insect population data failed to find consistent population trajectories (20). While several of these studies have received criticism for not including important contextual information in the analysis (23, 24), this perhaps highlights an essential caveat to attempts to synthesize insect population trends: without species-specific considerations and a firm grasp of underlying data structure, synthesis attempts are more likely to miss or incorrectly assign drivers of change (25). Thus, while generalizations may be useful to signal a greater problem, understanding the causes of, and solutions to, insect decline is unlikely to be realized without critical, well-informed place-based longitudinal analyses of particular taxonomic groups (26).

Given current uncertainties, a more refined understanding of insect declines is needed to develop meaningful loss mitigation strategies. Insects contribute to a wide variety of ecosystem services in working landscapes, especially pollination, plant growth regulation via herbivory, biological control of pest organisms, and decomposition (27). Functional losses in insect communities may result in dire economic and social consequences. Yet in agricultural systems, certain insects, especially exotic and invasive species, are predicted to increase under future environmental conditions. Yield losses in staple crops like rice, maize, and wheat are projected to increase by 10–25% per degree of global mean surface warming resulting from increased population growth of many pest species (28). Exotic predaceous insects, including those imported for biological control purposes, may, like invasive herbivores, be similarly favored by changing conditions due to invasibility-related traits, although population responses may be less predictable due to system-specific trophic dependencies (29). A recent study of herbivore–natural enemy interactions suggested invasive and pest species may outpace natural enemies under climate change conditions (30). These observations suggest that invasive insects may in fact defy general trends of decline observed among insects of conservation concern if insects with traits that confer invasibility are more resilient in the presence of drivers that lead to declines in the majority of insect species (31).

Lady beetles (Coleoptera: Coccinellidae) provide an ideal case study for understanding complex dynamics in insect communities. Of nearly 500 species known in North America (32), agricultural systems tend to support assemblages of 10–15 species varying in species composition by locality (33–35), with these assemblages generally seen as important contributors to the biological control of agricultural pests (36). The community of lady beetles in many agricultural systems includes both native and introduced species, and as many of these species offer similar ecosystem functions, these systems allow for an explicit evaluation of differential performance of native vs. exotic species over time. Net predation services across a guild of diverse predators are associated with body size, habitat preference, and hunting mode of the predators (37). Because of their importance in pest control, we and others have developed and field-validated methods for estimating biocontrol provision from guilds of natural enemies, making it possible to estimate ecosystem service capacity of the community from abundance measures alone (38–40). While it remains difficult to estimate realized ecosystem service provision at low prey population densities in particular due to density dependence and competition between community members (41), these approaches, which create a weighted metric of predator abundance based on body size and consumption rate metrics, estimate the “ceiling” of predation services delivered by a community (42). In this study, we leverage a three-decade experiment to examine how lady beetle abundance and predation services change over time and in annual and perennial habitats within the same landscape. Lady beetles were monitored weekly during the growing season every year from 1989 using standardized yellow sticky traps in a long-term row crop agroecosystem experiment with 10 plant community and management treatments (43). Data were examined to extract population trends using a combination of linear and nonlinear models. This allowed us to contrast rates of decline for native and exotic lady beetle species and communities, to determine the impact of habitat type on population trajectories and evaluate the impact on the community's capacity for biocontrol services over the full time series and selected shorter intervals.

## Results

### Long-term population trends

**Fig. 1.**
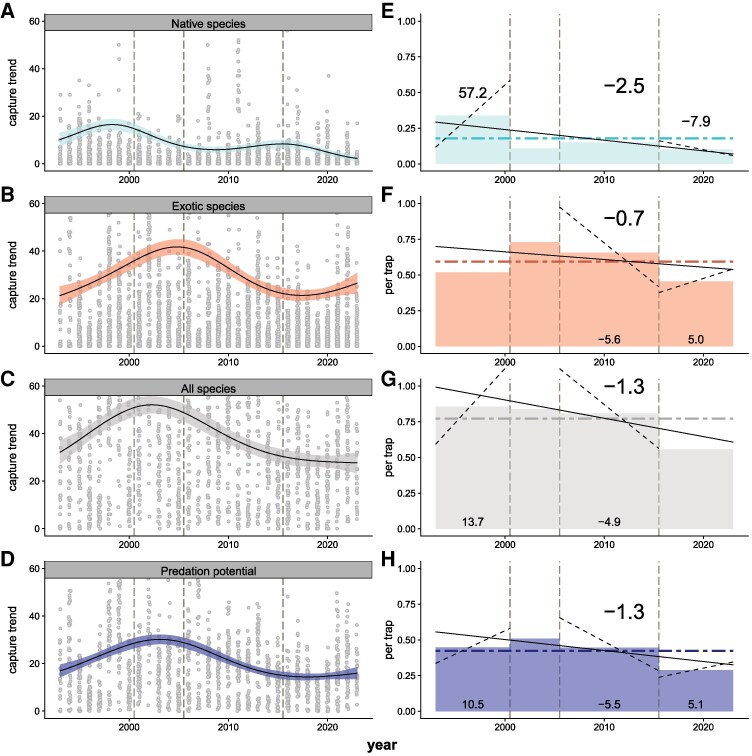
Coccinellid abundance and predation potential at the Kellogg Biological Station Long-Term Ecological Research site 1993–2023. A, E) Native species; B, F) exotic species; C, G) entire community; and D, H) predation potential (total abundance weighted by estimated average species consumption rate of aphids). A–D show raw captures with trajectories derived from GAMs estimating captures through time as a function of standardized sampling effort (50 traps per sample), with the SE of the estimate shown as a shaded ribbon. A–D are truncated at 60 observations per sample for visual clarity of the modeled trend. Dashed vertical lines on each panel correspond to established (2000, 2005) and hypothesized (post 2015) dynamical shifts in the community. E–H show linear trends over the same time period, presented in units of captures per trap. The horizontal dot-dash lines represent the mean captures per trap over the 31-year study period, while the solid black line gives the slope of a SLR of captures per trap by year. The large number in bold is the percentage change per year based on the overall regression. Within each dynamical period, the shaded rectangle represents the average captures per trap period, while the dashed line represents the slope of the captures per trap over time for significant trends. Numbers within each dynamical period represent the percent change per year when a statistically significant change was detected.

Over the 31 years of data included in the study, significant nonlinear patterns of abundance occurred in both native and exotic coccinellid species, the total community, and predation potential (Table 1, Fig. 1A–D). Native species abundance peaked in 1998 and again at a lower level around 2015 before exhibiting a steep decline since 2016 (Fig. 1A). Exotic species were overall more abundant than natives, peaking in 2005 and again in 2023 (Fig. 1B). The total community was influenced by these opposite trends, peaking in 2002 and then steadily declining since, with the increase in exotics since 2016 moderating the decline in the total community (Fig. 1C). Predation potential largely followed the abundance of exotic species (Fig. 1D).

**Table 1.** Long-term (1993–2023) and recent (2016–2023) trends in coccinellid abundance and predation potential at the Kellogg Biological Station Long-Term Ecological Research Site, Hickory Corners, MI.

Group	Total % change 1993–2023 (slope)	Total % change since 2016 (slope)	Nonlinear effect through time?	Crop type specific effect?
Native species	−76.6 (−0.007)	−62.9 (−0.01)	Yes (edf = 3.90, GCV = 12.8). Two peaks over time: increase to 1998 then a strong drop troughing near 2008, peak again near 2015 then drop, but overall decreasing.	Yes (GCV = 11.4). No temporal nonlinearity in forests, two peaks over time in annual and perennial crops, with pattern more pronounced in annual systems.
Exotic species	−23.1 (−0.005)	39.9 (0.02)	Yes (edf = 3.97, GCV = 19.9) Peaked distribution over time, peaking near 2007, with decline until 2018 and recent increase approaching historical mean.	Yes: slight (GCV = 19.6). Patterns largely track overall trends, except increase in annual crops in the most recent 5 years. No temporal nonlinearity in forests.
Total community	−38.8 (−0.01)	NS	Yes (edf = 3.98, GCV = 21.0). Increased until 2000, then declined at variable rates for the last 20 years.	Yes (GCV = 20.5). No temporal nonlinearity in forests, but domed distribution through time in annual and perennial croplands, both peaking near 2005.
Predation potential	−41.5 (−0.007)	40.5 (−0.01)	Yes (edf = 3.96, GCV = 11.9), domed distribution through time increasing to 2003–2004 and decreasing until 2018.	Yes (GCV = 11.7). Patterns largely track overall abundance.

Linear trends, given in net percent change over the three-decade study and recent dynamical phase (with yearly percent change given in parentheses) and GAM fits for lady beetle communities through time at Kellogg Biological Station. Nonlinearity was measured as a function of the magnitude of the effective degrees of freedom (edf > 1, where 1 approximates a linear fit through time and increasing edf indicates increasing nonlinearity through time in the trend). In all cases, significant (*P* < 0.05) nonlinear trends were detected for all lady beetle community metrics. Model selection using global cross validation (GCV, where a smaller GCV indicates a better fit) was then used to determine whether temporal structure differed between habitats (additional fit statistics are available in Table 2).

The long-term linear trends (1993–2023) for the abundance of native and exotic coccinellid species, the total community, and predation potential were consistently negative (Fig. 1E–H) with final values well below their long-term means (Table 1). Native species declined at a rate of 2.5%/year, a total of 76.6% over the entire period, and at an accelerated rate of 7.9%/year, totaling a 62.9% decline since 2016. Exotic species declined at 0.7%/year, a total of 23.1% over the full study, even while increasing 39.9% since 2016. The total community declined 1.3%/year, a total of 38.8% over the full study even though no linear trends in overall population size could be detected since 2016. Finally, predation potential declined 1.3%/year, totaling 41.5% over the full study while increasing 40.5% since 2016.

The selected dynamical time periods showed widely varying linear trends, illustrating the difficulty in reliably detecting population trajectories from shorter-term data sets. For example, native coccinellids (Fig. 1A and E) were strongly increasing at 57.2%/year from 1993 to 2000, appeared largely unchanged during the next two time periods, and decreased at 7.9%/year in the last 8 years. Yet, the net trend in this population over the 31-year time series was a marked decline averaging 2.5%/year. Linear trends estimated over shorter periods are highly sensitive to population sizes near the start and end of each interval, and the assumption of linear change can mask the cumulative effects of compounding. For example, the 57.2%/year rate of increase by native coccinellids observed during the first phase of observation is largely driven by a single year where a higher population was observed in 1998. Exotic coccinellids (Fig. 1B and F), the total community (Fig. 1C and G), and predation potential (Fig. 1D and H) also show fluctuating periods of increase or decrease ranging from 10.5 to −5.5%/year depending on the time period. In all cases, and regardless of trajectory, all groups had a lower-than-average overall capture rate in the most recent time period.

### Influence of habitat type

**Fig. 2.**
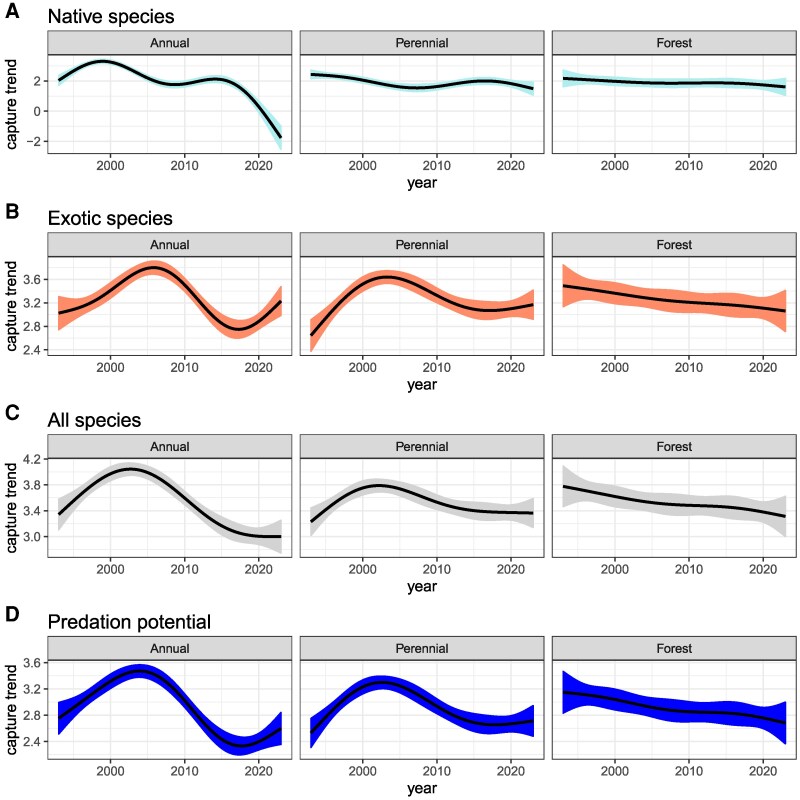
Coccinellid abundance and predation potential by habitat type (annual crops, perennial crops, and forest) at the Kellogg Biological Station Long-Term Ecological Research site 1993–2023. A) Native species only; B) exotic species only; C) entire lady beetle community; and D) predation potential (total abundance weighted by estimated average species consumption rate of aphids). Smoothed trajectories derived from GAMs estimating capture trends through time as a function of standardized sampling effort (50 traps per sample), with the SE of the estimate shown as a shaded ribbon.

For almost all taxa and aggregate measures, the abundance of coccinellids varied through time with differing patterns by habitat (Fig. 2). Variation was, in all cases, best explained by GAM models that partitioned variance by aggregated crop type (i.e. these models had the lowest value of the minimized generalized cross-validation [GCV] score associated with their fit compared with models that did not partition variance by crop type; Table 1). In all cases, significant nonlinear temporal structures were detected for metrics in annual and perennial croplands, but no nonlinear trends were detected in forests (i.e. the overall trend in time in forests could be approximated by a linear downward trend; Table 2).

**Table 2.** Long-term trends in coccinellid abundance and predation potential in three habitat types at the Kellogg Biological Station, Long-Term Ecological Research Site, Hickory Corners, MI.

Lady beetle group	Plant community	edf	Ref. df	*F*	Significant
Native species	Annual	3.74	3.96	78.259	*
	Perennial	3.77	3.97	7.736	*
	Forest	3.58	3.9	0.663	
Exotic species	Annual	3.93	3.99	23.933	*
	Perennial	3.93	3.99	13.461	*
	Forest	3.87	3.99	1.459	
Whole community	Annual	3.95	3.99	36.722	*
	Perennial	3.95	3.99	7.778	*
	Forest	3.9	3.99	1.845	
Predation potential	Annual	3.91	3.99	39.035	*
	Perennial	3.91	3.99	16.745	*
	Forest	3.82	3.98	2.133	

*Significant (*P*<0.05) nonlinear trends were detected for all lady beetle community metrics in annual and perennial croplands but not in forests.

Populations were more variable in annual crops compared with perennial crops, as reflected by higher *F* values indicating more structural variance being partitioned to annual crops within each model (Table 2). The temporal patterns of abundance of native species differed between habitat types, with very pronounced patterns of peaks in 1998 and periods of decline from 1998 to 2008 and 2016–present in annual cropping systems, weaker changes in perennial plots, and no significant temporal pattern found in forests (Fig. 2A). Exotic species underwent a rapid increase from the beginning of the study in both annual and perennial crops, peaking around 2004 in perennial and 2006 in annual crops (Fig. 2B). After these peaks, capture rates underwent a rapid decline in annual crops and slightly less pronounced decline in perennial crops. Temporal structure was not detected in forest plots for exotic species. The total community of coccinellids increased from 1993 until 2004–2005 in annual crops and then declined sharply before more recent moderation in the decline pattern (Fig. 2C). In perennial vegetation, the total community had a similar peak in 2004–2005 but then a more gradual decline. In forests, we observed a relatively uniform pattern of decline in the total community over the entire three-decade period, with no significant temporal variation in the trend (Table 2). When the community measurements were converted to predation potential (Fig. 2D), we observed a pattern that largely follows total abundance of exotic species, although extremes appear to be buffered slightly earlier in the study period when native species were more commonly captured.

### Individual species trends and influences

**Fig. 3.**
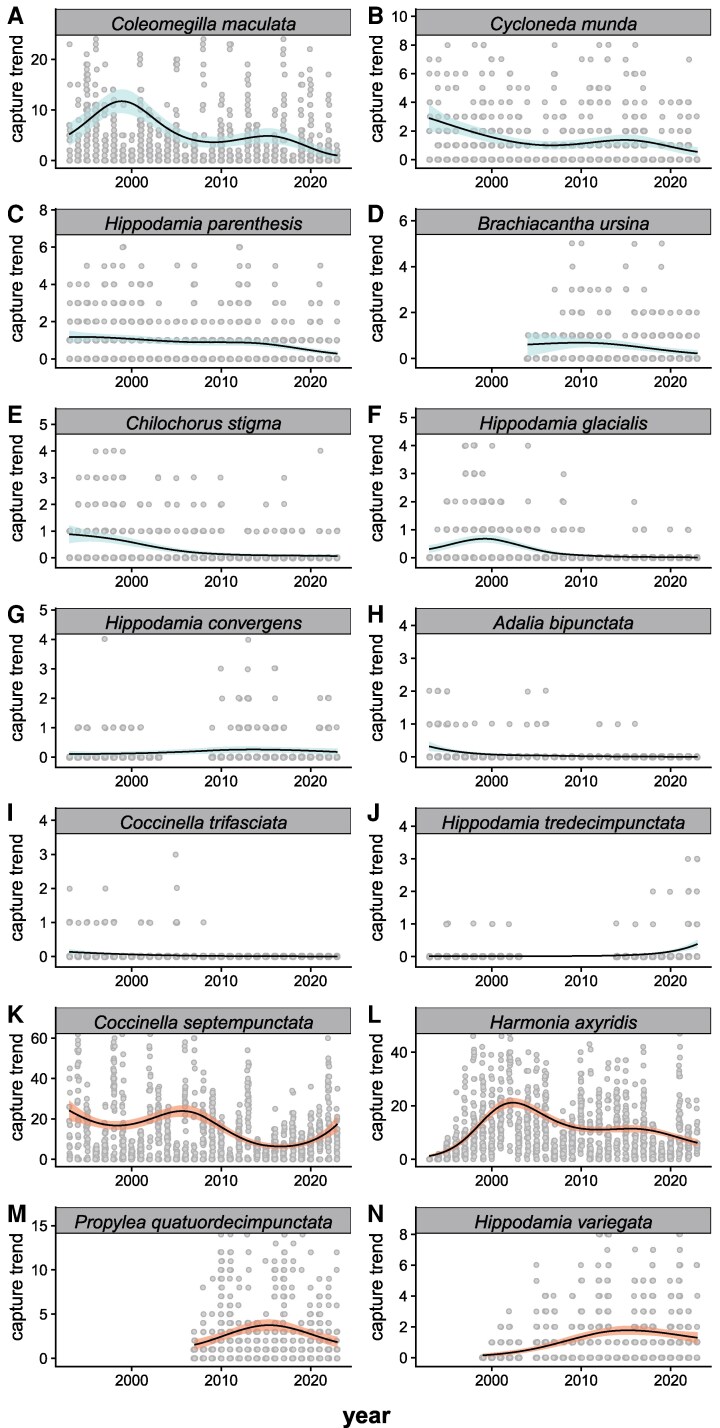
Coccinellid abundance by species at the Kellogg Biological Station Long-Term Ecological Research site 1993–2023. Panels show raw captures with trajectories derived from GAMs estimating captures through time as a function of standardized sampling effort (50 traps per sample), with the SE of the estimate shown as a shaded ribbon. Native species (A–J) are indicated with blue shading. Exotic species (K–N) are indicated with orange shading. Within each group, panels are ordered by total abundance as follows: native species A) *C. maculata*; B) *Cy. munda*; C) *H. parenthesis*; D) *B. ursina*; E) *Chilocorus stigma*; F) *H. glacialis*; G) *H. convergens*; H) *A. bipunctata*; I) *Co. trifasciata*; J) *H. tredecimpunctata*; and exotic species K) *Co. septempunctata*; L) *Ha. axyridis*; M) *P. quatuordecimpunctata*; and N) *H. variegata*. The *y*-axes are truncated at 5× the mean capture rate for each species for visual clarity of the modeled trend. Additional information on GAM fits, linear trends, and collection details is available in Table S2.

Individual species exhibited unique population trends that inform the overall pattern of abundance and predation potential. Of the 10 native species, *Coleomegilla maculata* heavily influenced the long-term population trends (Fig. 3A). This abundant species exhibits a population trend that largely drives the overall native community trend, including the steep recent decline. In the last 8 years, *C. maculata* has declined 2.2%/year or −17.8% explaining about half the total decline in natives during this time period (Table S2). Of the remaining nine native species, eight are in long-term decline (Fig. 3, Table S2). *Coccinella trifasciata* and *Adalia bipunctata* have not been observed since 2008 and 2016, respectively. Three formerly common species, *Chilocorus stigma*, *Hippodamia glacialis*, and *Hippodamia convergens*, have fallen below detection limits in some of the last 5 years. The only exception is the rarest species at the site, *Hippodamia tredecimpunctata*, which has been observed more frequently in the past 6 years after a period when it was not detected.

Of the four exotic species at the site, *Coccinella septempunctata* has been present since the start of sampling in 1993, while *Harmonia axyridis*, *Propylea quatuordecimpunctata*, and *Hippodamia variegata* were first recorded in the survey in 1994, 2007, and 1999, respectively. Both *Co. septempunctata* and *Ha. axyridis* contribute to the overall abundance patterns at the site. *Coccinella septempunctata* has been the overall most abundant species at the site with peaks in 1993 and 2005. Since 2016 this species has been increasing at 19.5% per year (Table S2). In contrast, *Ha. axyridis* peaked in 2002 and 2016 and has since been nearly stable in the last 8 years. *Propylea quatuordecimpunctata* and *H. variegata* both reached peaks in 2015–2016. While *H. variegata* has been linearly stable in the past 8 years, *P. quatuordecimpunctata* is currently declining in abundance at a rate of 5.7%/year (Table S2). For *Ha. axyridis*, *P. quatuordecimpunctata*, and *H. variegata*, GAM analysis suggests recent decreasing trajectories in capture rates.

## Discussion

Taken together, the overall trend of lady beetles in this system is one of marked decline over our 31-year study. Insect decline has been described as a “death by 1,000 cuts,” with many drivers interacting nonuniformly to produce overall trends of biodiversity loss (21). Our long-term study supports this description. Even within a single locality, drivers of insect population dynamics may vary dramatically over time. We found that lady beetle population trajectories varied over time and exhibited differential patterns over habitat types and that native and exotic species experienced asynchronous periods of decline.

As evidence of the shifting dynamics of beetle abundances, the results of this study stand in contrast to a study completed a decade ago in the same system. In previous work ending in 2015, we observed a period of loss and then stabilization in native species after the arrival of three exotic lady beetle species (44). At that time, we concluded that the establishment of *Ha. axyridis*, in particular, had been facilitated by the accidental introduction of its preferred prey, *Aphis glycines*, from its native range (45), but that perennial habitats and forests appeared to buffer these effects, and management of *A. glycines* would buffer further loss of native lady beetles. However, since 2016, we have observed the apparent emergence of a new dynamical period in this community, with an inflection in the trajectory of native species from one of relative stability to steep decline, taken together with a period of overall stability of exotic species.

An important finding of this work is that exotic species and are, in fact, declining at a rate similar to native species at this site, although asynchronously at shorter time scales. While one exotic species, *Co. septempunctata*, has recently been observed increasing (possibly associated with recent warm conditions in spring, when this species is most active (46)), the net community size of exotic species is currently well below its long-term average, and populations of other exotic species are either stable or declining. While we have noted in the past that exotic species have been successful in replacing the lost predation ecosystem services at this site due to native species loss (39), we have also noted that the resilience of this service is imperiled when it is not delivered by a diverse community with functional redundancy (47). This creates a fundamental risk to the stability of the system, particularly in a changing environment (48). Although we do not specifically examine environmental drivers of change in this study, previous work has attributed climate variation as a driver of the changing dominance of species within the non-native lady beetle guild: *Co. septempunctata* may be more specific in its environmental tolerances than even other exotic lady beetle species (46).

Multiple drivers have been implicated in declines in invaded lady beetle communities. Although direct competition with non-native species is often associated with population declines of native species (49), several studies have questioned the assumption that exotic species act alone as a driver of native species loss (50, 51) and that these drivers instead interact with individual species responses to landscape, climate, and management. Landscape structure appears to play a strong role in the structuring of lady beetle communities in the US Midwest (52, 53) and directly impacts the ability of insects in these landscapes to deliver ecosystem services (54–56). In long-term co-existing communities of native and exotic lady beetles, spatiotemporal niche partitioning has been observed between resident exotic and native guilds (57) and even between two individual exotic species (46). These varied niches, however, slight, contribute to uneven responses to interacting drivers of population responses that we observe in this system.

The population trends we observed are not static, suggesting that the drivers themselves are also changing over time. While we can generally attribute the dynamics we observed in this community until 2016 to known changes in agricultural management (45) or invasions of adventive lady beetles and prey (44, 58), we note that no novel exotic species nor major landscape changes occurred at the site between 2016 and 2023. Yet, a net decrease in the abundance of exotic lady beetles in the community over our study period suggests that conditions at the site have not remained consistently favorable. Possible explanations for this trend amongst exotic species include the increasing frequency of brief periods of temperature extremes affecting even species more capable of capitalizing on warming conditions (46, 59) or the continued use of neonicotinoid seed treatments affecting prey availability at the landscape level (17, 60).

Although the lady beetle community exhibited a general trend of decline throughout the last three decades, we note that trends are not uniform across habitats. In general, although lady beetles were most frequently captured in annual cropping systems, perennial crops and forests considerably buffered shorter-term fluctuations in their populations. Annual cropland habitats undergo numerous disturbances, both within (e.g. planting, management, and harvest) and between (e.g. crop rotation) growing seasons. Perennial vegetation offers a greater stability of resources to a wide range of beneficial organisms (61–63), and landscapes that incorporate more perennial and forested habitat are associated with greater biocontrol services conferred by lady beetles (53, 54). Habitat patches with less disturbance may act as a refugia for native and exotic insects alike; however, population trends in these patches represent a best-case scenario (64). Long-term monitoring schemes like ours are sensitive to an insidious bias: for monitoring to continue at a site over decades, it is contingent on land use patterns to stay relatively consistent so that sampling can continue consistently. Yet, anthropogenic change, including land use and management change, is more the norm than the exception (7). When insect populations are demonstrably declining (as we observed) in relatively stable portions of a landscape specifically managed as a long-term experiment and conservation reserve, the patterns likely represent a conservative estimate (64).

Future studies of prey abundance and predation rates in differing crops/habitats may further elucidate drivers of change in predation potential. Actualized pest suppression rates of aphidophagous Coccinellidae are highly complex and difficult to measure directly because many of these species will switch between appropriate prey and display varied density-dependent functional responses to prey items, and intraguild interactions between community members may further modify these rates (65, 66). While our model of predation potential places an upper bound on the impacts of predation, additional approaches may be helpful in pointing to current drivers of change.

## Conclusions

Lady beetles are essential biological controls in many agricultural systems. Their decreasing overall abundance coupled with a lack of biodiversity preservation in this community presents a serious threat to the stability of ecosystem services. However, drivers will vary over time; even within a single taxonomic group, there is unlikely to be a single cause acting consistently that explains insect biodiversity loss. We have demonstrated that even among a group of closely related species with similar ecological functions, rates of change and apparent drivers differ between species, in different parts of the landscape, and with time. Recent dynamics in the lady beetle community in our region suggest two important points. Firstly, even exotic species, presumed to have traits that may confer more resilience to changing environments, have exhibited overall declines over the last three decades. Secondly, while perenniality, in crops and forest, offered some resistance to short-term fluctuations in insect populations, lady beetles in these habitats are also in decline. Continued study of these patterns coupled with measurement of prey abundance and predation rates will be helpful in resolving which drivers dominate at any point in time.

## Materials and methods

Lady beetles were monitored annually in seven plant community treatments since 1989 at the Kellogg Biological Station, Long-Term Ecological Research Site, Main Cropping Systems Experiment, Hickory Corners, MI, United States (Table S1). In 1993, three additional mid-to-late successional forest habitats were added to the survey. In all habitats, lady beetles are collected on yellow sticky traps deployed weekly during the growing season (67). Here, we use data collected from all 10 plant communities for every year from 1993 to 2023. For analysis, data were aggregated across habitats in two different ways: first, as all captures regardless of habitat, and then aggregated by crop type. Annual crops included rotations of corn, soybean, and wheat under varying intensities of management; perennial crops included alfalfa and switchgrass grown as forage, poplar plantations, and early successional vegetation; and finally, forests, which included conifer plantations, mid-successional forests on former cropland, and old-growth deciduous forests.

Data were culled to remove observations taken prior to 1993, and within each year, data collected after day-of-year 222 (August 10) were removed to provide an approximately equal sampling period each year. Data were aggregated as the total number of adult lady beetles of a given species captured by treatment by replicate (*n* = 6), per year (*n* = 31), and sampling effort was quantified as the number of traps reporting data for a given taxon in that same treatment by replicate combination within each sampling year (i.e. summing across subsamples and sampling weeks). We computed the total number of captures from native species; *A. bipunctata*, *Brachiacantha ursina*, *Chilocorus stigma*, *Co. trifasciata*, *C. maculata*, *Cycloneda munda*, *H. convergens*, *H. glacialis*, *Hippodamia parenthesis*, *H. tredecimpunctata*, captures of exotic species; *Co. septempunctat*a, *Ha. axyridis*, *H. variegata*, and *P. quatuordecimpunctata*, and all captures combined. We then computed the total community predation potential, a measurement where the number of individuals of each lady beetle species captured in that treatment by replicate by year combination was multiplied by a weighting factor corresponding to the number of aphid prey that species has been observed eating in a day, divided by 100, then summed (after 39).

### Trajectory analysis

For each taxon and aggregate measurement, we conducted several statistical analyses to assess changes to the observed population over time. We used simple linear regression (SLR) to compute overall linear trends in captures per trap and average rates of change over time, where percent change per year was calculated as the difference between the modeled mean capture rate at the start and end of each observation period (estimated from the line of best fit), divided by the number of years in the period. While linear models fit a straight line to the data, providing intuitive outputs that are useful in estimating overall rates of change, in data produced by complex systems, rates of change are often nonuniform and vary in space and as they interact with other processes. Therefore, we also used generalized additive models (GAMs) (68), which enable the fitting of nonlinear and complex relationships driven by environmental variation through time by computing localized trends in the data, where model predictions are more influenced by variables with values near to what is being predicted than those farther from them (69).

GAMs were fitted using mgcv (70). GAMs used the total number of captures within a sampling period as a function of a smoother on year, constrained with a smoothing parameter *sp* = 0.5 to minimize overfitting. The function was also constrained so that the number of knots could not exceed one for every 6 years of data available (=5, for taxa reported over the entire study period, but fewer for species recorded for shorter periods). Sampling effort was incorporated into the model by including an offset composed of the natural log of number of traps. A quasi-Poisson error structure was used to fit all models. For visualization of GAM functions, the model fit was used to predict capture rates over the sampling period using a standard sampling effort of 50 traps per year within the sampling unit, and this response was re-projected in the natural response range of the data. To determine whether temporal structure varied by plant community, treatment groups (treatments aggregated into annual crops, perennial managed plant communities, and forests) were encoded as categorical terms interacting with year. We used the GCV, the global cross-validation statistic, to determine which models had better fit (where lower GCV indicates a better fitting model) when grouped by plant community. In this case, we interpreted that if the model containing a plant community term had a better fit, that this was evidence that population patterns through time differed between plant communities. In the interpretation of individual temporal trajectories (i.e. the pattern fit to an individual lady beetle species in an individual plant community through time), we used statistical significance (a *P*-value of <0.05) to determine “significant structure through time”; the effective degrees of freedom to assess the “wiggliness” (corresponding to the underlying number of functions required to construct the curve of best fit), and the *F* statistic to assess the relative variation partitioned into each relationship compared with the variation in other plant communities.

For linear analysis, we also conducted SLR on time periods corresponding to known dynamical phases associated with populations of *Ha. axyridis*, one of the dominant exotic species at the site (45, 71). The dynamical periods included phase I, 1993–2000, the initial establishment period of this species; phase II, 2001–2005, the invasion and outbreak of soybean aphid, a preferred prey item for lady beetles; and phase III, 2006–2015, a period of stabilization as insecticidal control of soybean aphid became widely adopted. We also included a phase IV, for data taken after 2015, based on an apparent inflection in observed dynamics in native species (see Results).

## Supplementary Material

pgag063_Supplementary_Data

## Data Availability

All data manipulation and aggregation, as well as all exploratory and analytical approaches, were completed in R. The analysis code and development history are available as an archived GitHub repository (72). Organismal data sets (67) utilized for this research are mirrored within our GitHub repository as CSV files.
