# Supplementary material for: Asynchronous declines of native and exotic insect predators reduce pest suppression potential in agriculture
Source: PNAS Nexus. 2026 Mar 10;5(3):pgag063. doi: 10.1093/pnasnexus/pgag063 (PMC13001591; doi:10.1093/pnasnexus/pgag063)
Supplement: pgag063_Supplementary_Data [file pgag063_supplementary_data.docx]

**Supplemental Information for: Bahlai and Landis, Asynchronous declines**

**Table S1**. **Description of treatments sampled for Coccinellidae in the KBS LTER Main Cropping System Experiment (MCSE)** ^A^. Reformatted from Robertson and Hamilton 2015

| **Cropping Systems/ Community** | **Management Practices** |
| --- | --- |
| **Annual Cropping Systems**  Conventional (T1) | Prevailing norm for tilled corn–soybean-winter wheat (c–s–w) rotation; standard chemical inputs, chisel-plowed, no cover crops, no manure or compost |
| No-till (T2) | Prevailing norm for no-till c–s–w rotation; standard chemical inputs, permanent no-till, no cover crops, no manure or compost |
| Reduced Input (T3) | Biologically based c–s–w rotation managed to reduce synthetic chemical inputs; chisel-plowed, winter cover crop of red clover or annual rye, no manure or compost |
| Biologically Based (T4) | Biologically based c–s–w rotation managed without synthetic chemical inputs; chisel-plowed, mechanical weed control, winter cover crop of red clover or annual rye, no manure or compost; USDA-certified organic |
| **Perennial Managed Communities**  Poplar (T5) | Hybrid poplar trees on a ca. 10-year harvest cycle, either replanted or coppiced after harvest |
| Alfalfa/Switchgrass (T6) | 5- to 6-year rotation with winter wheat as a 1-year break crop changed to switchgrass 2017 |
| Early Successional (T7) | Historically tilled cropland abandoned in 1988; unmanaged but for annual spring burn to control woody species |
| **Forests**  Mid-successional (SF) | Historically tilled cropland abandoned ca. 1955; unmanaged, with regrowth in transition to forest |
| Coniferous Forest (CF) | Planted conifers periodically thinned |
| Deciduous Forest (DF) | Late successional native forest never cleared (two sites) or logged once ca. 1900 (one site); unmanaged |

^A^Treatment codes that have been used throughout the project’s history are given in parentheses.

**Table S2. Long-term (1993-2023) and recent (2016-2023) trends in coccinellid abundance by species at the Kellogg Biological Station, Long Term Ecological Research Site, Hickory Corners, MI.** Linear trends, given in net percent change over the three-decade study and recent dynamical phase (with yearly percent change given in parentheses), and generalized additive model fits for lady beetle communities through time. Non-linearity was measured as a function of the magnitude of the effective degrees of freedom (*edf*>1, where 1 approximates a linear fit through time and increasing *edf* indicates increasing nonlinearity through time in the trend). Model selection using global cross validation (GCV, where a smaller GCV indicates a better fit) was then used to determine if temporal structure differed between habitats. Non-significant trends are denoted NS.

| **Species** | **Avg per 50 traps** | **Detection frequency 2019-2023 (year last observed)** | **Total % change 1993-2023 (slope)** | **Total % change since 2016 (slope)** | **Non-linear effect through time? Evidence of differential effects by crop type?** | **Notes** |
| --- | --- | --- | --- | --- | --- | --- |
| *Coleomegilla maculata* | 5.70 | 5 | -83.7  (-0.006) | -17.8  (-0.007) | Yes (edf=3.83, GCV=14.4). Very strong temporal dependency, very cyclic looking overall pattern but general decline. Pattern varies by crop type (GCV=12.2), with significant temporal variability observed in annual and perennial crop plots, but pronounced decline in annual in recent years |  |
| *Cycloneda munda* | 1.38 | 5 | -60.0  (-0.0007) | -95.4  (-0.006) | Yes (edf=3.53, GCV=3.2). Decreasing trends stabilized in mid-2000s, then increased from 2010-2015, but rapidly dropped after 2015. Dynamic was most pronounced in perennial cropland, but model performance does not improve when temporal variation is partitioned by crop type (GCV= 3.2) |  |
| *Hippodamia parenthesis* | 0.86 | 5 | - 59.6  (-0.0005) | -65.5  (-0.0015) | Yes (edf=3.33, GCV=1.8). Fairly consistently common through most of study period, with a trough between 2000 and 2012, but apparent steep decline in recent years of study. Annual and perennial crops follow the same temporal pattern, with a sharper decline in annual plots in recent years, but no temporal structure within forest (GCV=1.7) | Was not monitored in forest plots 2004-2011 |
| *Brachiacantha ursina* | 0.50 | 5 | -38.5  (-0.0006) | -65.1  (-0.0012) | Yes (edf=1.72, GCV=1.7) Domed abundance through time when data aggregated across site. Crop type specific temporal structure does not improve model fit (GCV=1.7). | Species was added to the survey in 2004 |
| *Chilocorus stigma* | 0.31 | 4 (2023) | -91.7  (-0.0004) | NS | Yes (edf=2.72, GCV=1.0). Species persists at site but the trend suggests loss occurring around early 2000s and no real recovery. Species is primarily associated with perennial crops and trend is driven by observations from those plots (GCV=0.9). |  |
| *Hippodamia glacialis* | 0.25 | 2 (2022) | -2.7  (-0.0004) | ~-100%  (0.0002) | Yes (edf=2.30, GCV=0.8). Abundance peaks around 2000, then declines to occasional observations after 2010. Model performance is not improved by including crop type (GCV=0.8). | Was not monitored in 2004-2014 in forest plots |
| *Hippodamia convergens* | 0.18 | 3 (2023) | NS | NS | No significant non-linear effects detected (edf=2.10, GCV=0.9) | Species is relatively rare at site, removed from data sheet 2004-2008. |
| *Adalia bipunctata* | 0.07 | 0 (2016) | -74.4  (-0.0001) | - | Yes (edf=1.64, GCV=0.3). Strong decline curve with species becoming very rare within the first five years of study. No strong evidence of crop-type effects (GCV=0.3) |  |
| *Coccinella trifasciata* | 0.04 | 0 (2008) | -49.0  (-0.00007) | - | Yes (edf= 1.49, GCV=0.2). Temporal structure suggests decrease from rare to non-existent. It has been relatively rare since the beginning of study, with a handful of individuals captured across site each year, but has not been seen since 2008. No strong evidence of crop-type effects (GCV=0.2) |  |
| *Hippodamia tredecim-*  *punctata* | 0.008 | 3 (2023) | 293  (0.0001) | 161  (0.002) | Yes (edf=1.40, GCV=0.3). Species was very rare at the beginning of study and is now detected fairly consistently. No evidence of crop-type specific temporal structure (GCV=0.3) | Species is rare at site, was removed from data sheet from 2005-2013 but has been detected relatively often in 2022-2023. |
| *Coccinella septem-punctata* | 15.65 | 5 | -59.1  (-0.009) | 157  (0.03) | Yes (edf=3.95, GCV=20.5). Strong nonlinear trend with overall decline over time, steepest decline from 2007-2015 but increase in recent years towards historical average density. Pattern is driven by abundance in annual and perennial crops: this species is relatively rare in forest plots (GCV=19.8) | First recorded at site in 1985, present and abundant at the start of the study. |
| *Harmonia axyridis* | 11.58 | 5 | NS | NS | Yes (edf=3.91, GCV=8.7). Increasing after introduction to ~2000, then largely stable, then precipitous decline with inflection point near 2015. Patterns are fairly consistent between annual and perennial crops, but no temporal structure was detected in forests (GCV=8.2). | First recorded in 1994 |
| *Propylea quatuorodecim-punctata* | 2.76 | 5 | NS | -45.8  (-0.01) | Yes (edf= 1.96, GCV=5.0). It increased from 2007-2014 and then declined. This species is most common in perennial croplands, and the overall pattern is driven by temporal structure in these plots (GCV=4.8). | Species first recorded in survey in 2007 |
| *Hippodamia variegata* | 1.20 | 5 | 750  (0.001) | NS | Yes (edf=2.64, GCV=2.7). After the first detection at the site in 2000, it increased until 2013 and has declined since. This species is rare in forests and patterns are driven by captures in annual and perennial croplands (GCV=2.5). | Species first added to survey in 1999, no data in 2004 |
